# ROS1-rearranged putative lung adenocarcinoma presenting as carcinoma of unknown primary site: a case report

**DOI:** 10.18632/oncotarget.26233

**Published:** 2018-10-16

**Authors:** Masaya Taniwaki, Masahiro Yamasaki, Koto Kawata, Kazuma Kawamoto, Kunihiko Funaishi, Yu Matsumoto, Naoko Matsumoto, Nobuyuki Ohashi, Noboru Hattori

**Affiliations:** ^1^ Department of Respiratory Disease, Hiroshima Red Cross Hospital and Atomic-bomb Survivors Hospital, Naka-ku, Hiroshima, Japan; ^2^ Ohashi Clinic, Naka-ku, Hiroshima, Japan; ^3^ Department of Molecular and Internal Medicine, Institute of Biomedical and Health Sciences, Hiroshima University, Minami-ku, Hiroshima, Japan

**Keywords:** ROS1 rearrangement, putative lung adenocarcinoma, carcinoma of unknown primary site, oncogene, crizotinib

## Abstract

Carcinoma of unknown primary site (CUP) is diagnosed only in 2-9% of all cancer cases. Adenocarcinomas account for approximately 60% of CUP, and some of these are putative lung adenocarcinomas. The frequency of driver oncogene positivity in the putative lung adenocarcinomas is unknown, and the efficacy of targeting therapies for the driver oncogene is also unknown. This is the first case report of C-ros oncogene 1 (ROS1)-rearranged putative lung adenocarcinoma presenting as CUP showing a good response to ROS1 inhibitor therapy. A 55-year-old woman presented with neck lymphadenopathy. Computed tomography and [18F]-fluorodeoxyglucose (FDG) positron emission tomography (PET) showed swelling of the bilateral supraclavicular, left accessory, mediastinal, and abdominal lymph nodes. The pathological analysis of the lymph node specimen biopsy indicated adenocarcinoma with cytokeratin 7 and thyroid transcription factor-1 positivity. Thus, this case was identified as ROS1- rearranged putative lung adenocarcinoma presenting as CUP. Oral crizotinib, an ROS1 inhibitor, was administered at a dose of 250 mg twice daily. Four weeks later, several swollen nodes showed marked improvement, and eight weeks later, FDG PET showed almost no uptake. In conclusion, putative lung adenocarcinoma presenting as CUP may involve ROS1 rearrangement, and ROS1 inhibitor therapy may be effective.

## INTRODUCTION

Carcinoma of unknown primary site (CUP) is diagnosed only in 2-9% of all cancer cases [1]. CUP patients with a predictable primary site have a good prognosis. To determine primary sites, CUP have been categorized according to serum tumor markers, metastatic sites, histopathological structures, and immunohistochemical examinations [2, 3]. Adenocarcinomas account for approximately 60% of CUP [2] and are generally immunohistochemically positive for cytokeratin (CK) 7, thyroid transcription factor (TTF)-1, or napsin A and negative for CK 20. CUPs are generally presumed to be lung adenocarcinomas and are therefore treated as primary lung adenocarcinomas in clinical practice [3]; however, the frequency of driver oncogene positivity in putative lung adenocarcinomas is unknown. C-ros oncogene 1 (ROS1) rearrangement is a driver oncogene in lung cancer [4]. It occurs in approximately 1% of patients with non-small cell lung carcinoma (NSCLC), and treatment with ROS1 inhibitors prolongs progression-free survival [4]. However, to our knowledge, there is no case report of a putative lung adenocarcinoma with ROS1 rearrangement treated with ROS1 inhibitor therapy.

Here, we report a case of ROS1-rearranged putative lung adenocarcinoma presenting as CUP showing a good response to ROS1 inhibitor therapy.

## CASE REPORT

**Figure 1:**
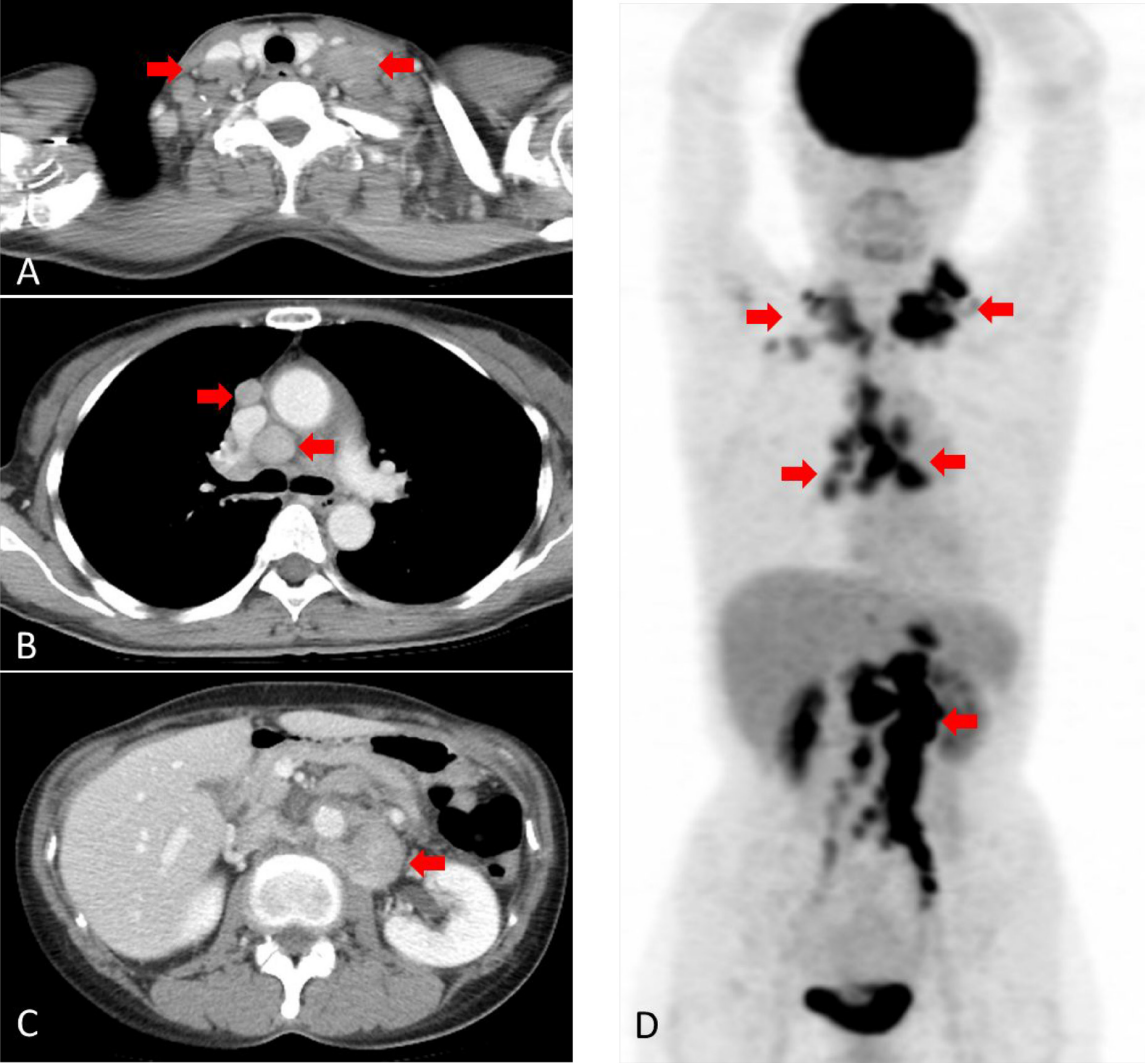
Computed tomography (CT) and [18F]-fluorodeoxyglucose (FDG) positron emission tomography (PET) before treatment (**A**) CT scan showing swelling of bilateral neck lymph nodes (arrowhead). (**B**) CT scan showing swelling of the mediastinal lymph nodes (arrowhead). (**C**) CT scan showing swelling of the abdominal lymph node (arrowhead). (**D**) FDG-PET scan showing high FDG uptake at the same lymph nodes detected via CT (arrowhead).

**Figure 2:**
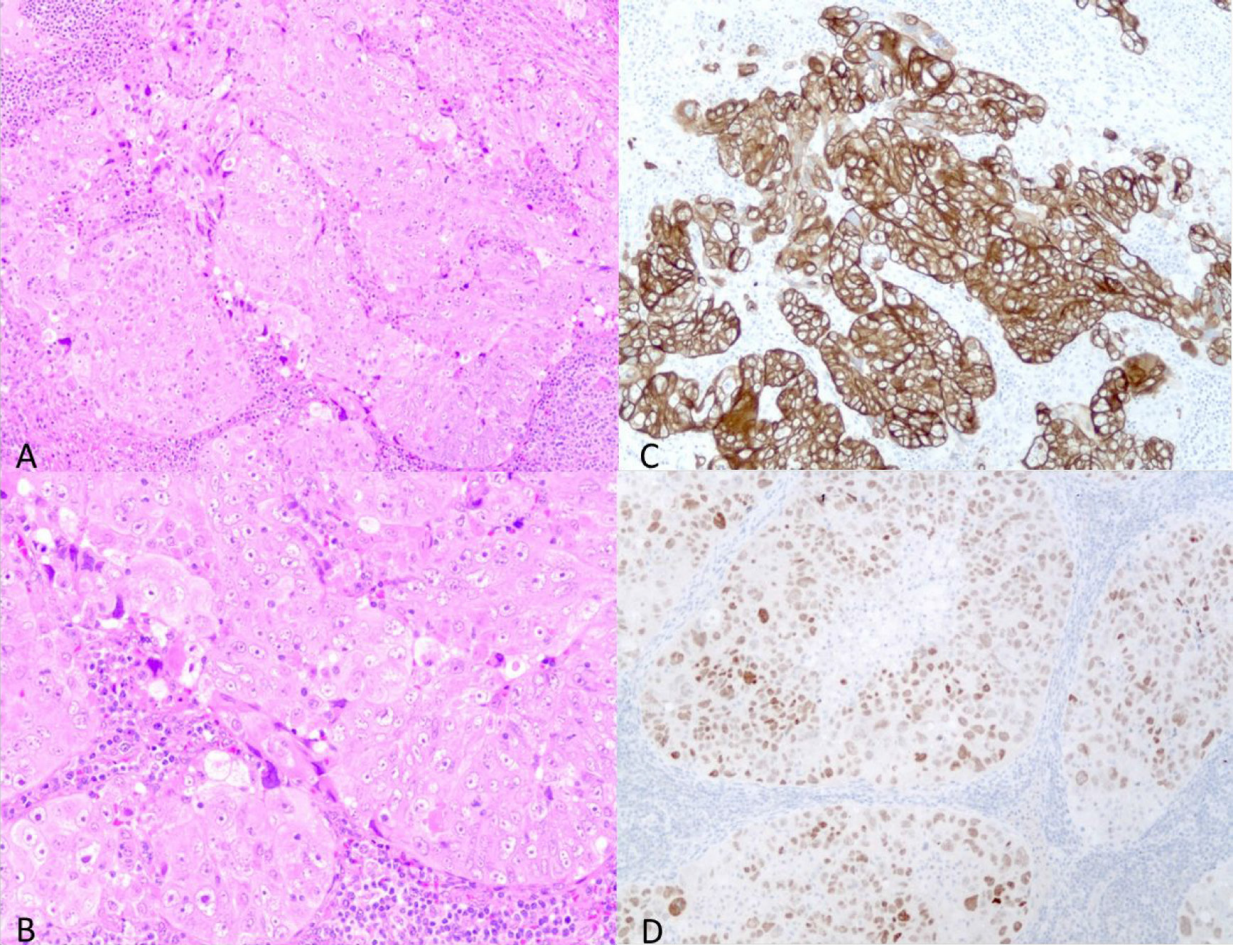
The hematoxylin-eosin (HE) and immunohistochemical staining of the left neck lymph node specimen (**A**) Low power view of the HE staining (A) and high power view (**B**). The structure of tumor cells showed that the tumor was an adenocarcinoma. The tumor cells showed CK7 positivity (**C**) and TTF1 positivity (**D**). These findings suggested that the tumor was a lung adenocarcinoma.

A 55-year-old woman with no smoking history presented to a hospital with chief complaints of bilateral lymphadenopathy of her neck. She had a panic disorder, and her family history was as follows: her father had liver cancer and mother had type 2 diabetes mellitus. On physical examination, swollen lymph nodes were palpable on both sides of her neck. Neck, chest, and abdominal computed tomography (CT) examination was performed, and swelling of the bilateral supraclavicular, left accessory, mediastinal, and abdominal lymph nodes were detected (Figure 1). She underwent [18F]-fluorodeoxyglucose (FDG) positron emission tomography, and high FDG uptake was detected at the same lymph nodes detected via CT examination. However, the primary site of the tumor could not be determined. Malignant lymphoma was suspected, and she was transferred to our hospital. The levels of each of the following markers were increased: serum squamous cell carcinoma (SCC) antigen, cytokeratin 19 fragments (CYFRA 21-1), carbohydrate antigen (CA) 125 (CA125), CA15-3, and soluble interleukin-2 receptor levels (36.7 ng/ml, 8.1 ng/ml, 1547 U/ml, 63.3 U/ml, and 1366 U/ml, respectively). We performed a neck lymph node biopsy, and histopathological examination showed that the tumor was a poorly differentiated adenocarcinoma. To detect the primary lesion of the tumor, she underwent upper gastrointestinal endoscopic examination, colonoscopy, and gynecologic examination; however, no primary site of the tumor was detected. Immunohistochemical staining of the left neck lymph node specimen showed CK7 and TTF-1 positivity (Figure 2). The results of the immunohistochemical staining led to the presumption that the primary site of the carcinoma was the lung or thyroid. The tumor specimen was also examined as an advanced primary lung adenocarcinoma and assessed for the following tumor markers: epidermal growth factor receptor (EGFR) mutation, anaplastic lymphoma kinase (ALK) rearrangement, ROS1 rearrangement, and programmed death-ligand 1 (PD-L1) expression. PD-L1 expression was examined by immunohistochemical staining with 22C3 antibody. As a result, ROS1 rearrangement and PD-L1 positivity (tumor proportion score [TPS]: 100%) were detected.

**Figure 3:**
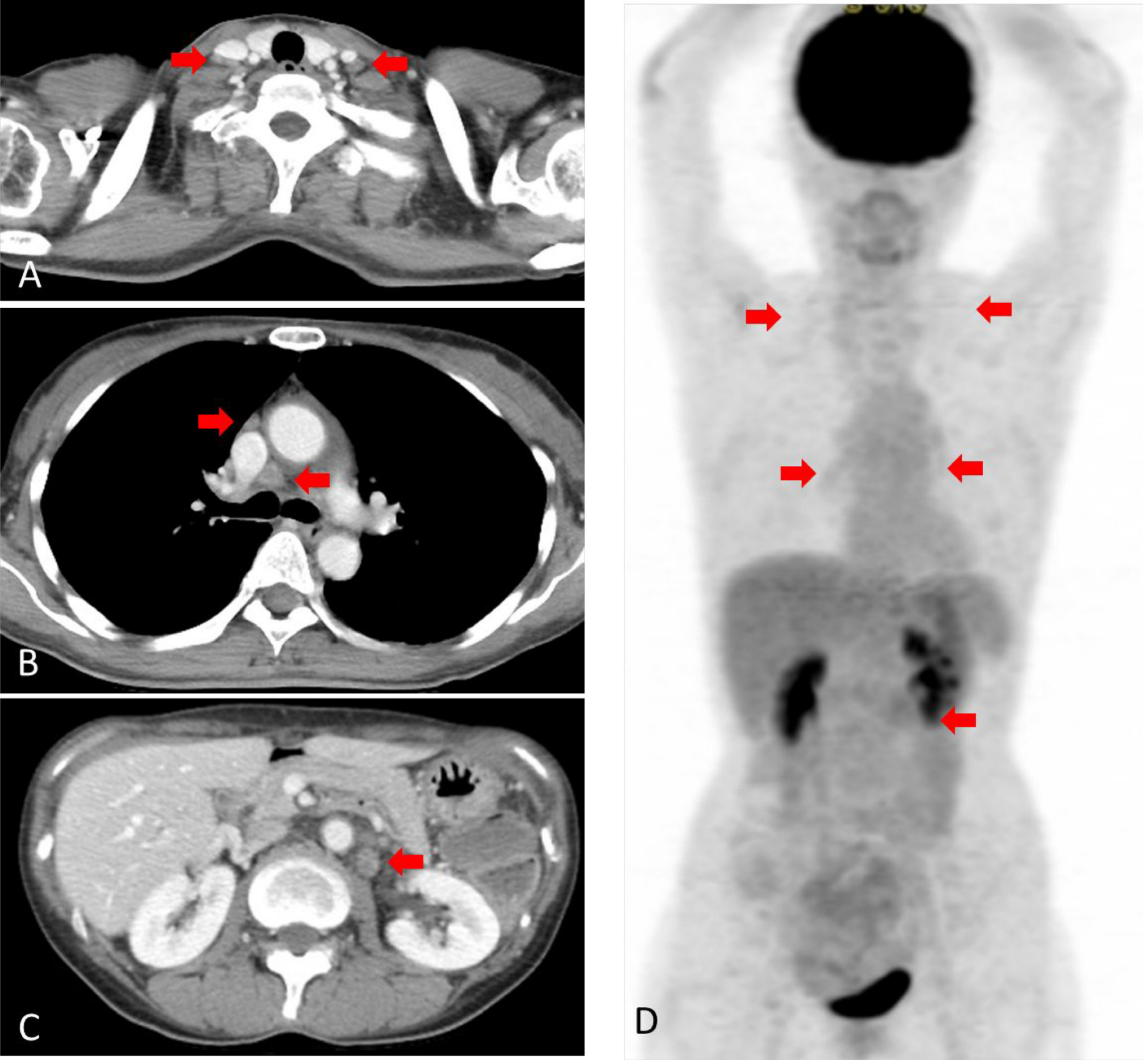
Computed tomography (CT) and [18F]-fluorodeoxyglucose (FDG) positron emission tomography (PET) after treatment (**A**) CT scan showing no swelling of the neck lymph node. (**B**) CT scan showing no swelling of the mediastinal lymph node. (**C**) CT scan showing no swelling of the abdominal lymph node. (**D**) FDG-PET scan showing no significant uptake.

Oral crizotinib, an ROS1 inhibitor, was administered at a dose of 250 mg twice daily. Four weeks later, the patient experienced crizotinib-related adverse events, including palsy of the whole body. Therefore, we reduced the dose of crizotinib to 250 mg once daily. Eight weeks later, all swollen lymph nodes showed marked improvement on CT examination and FDG PET (Figure 3). Serum SCC antigen, CYFRA 21-1, CA125, and CA15-3 levels also decreased remarkably (1.3 ng/ml, 1.7 ng/ml, 17 U/ml, and 15.7 U/ml, respectively). To date, the patient is alive with no disease progression and has continued crizotinib for a total of 3 months.

Informed consent was signed by the patient.

## DISCUSSION

In the present case report, we demonstrated two important clinical observations. First, putative lung adenocarcinoma presenting as CUP may have ROS1 rearrangement. In one study that identified CUP with no presumable primary site, ROS1 rearrangement was detected in 1 of the 200 (0.5%) cases of the study population [1]. Meanwhile, 6 cases of putative lung cancers were examined, but no ROS1 rearrangement was detected [5]. The frequency of ROS1 rearrangement in putative lung adenocarcinomas is estimated to be low; however, driver oncogenes, including ROS1 rearrangement, should be examined to provide appropriate treatments for patients.

Second, ROS1 inhibitor therapy may be effective for ROS1-rearranged putative lung adenocarcinoma. To our knowledge, this is the first case report of ROS1-rearranged putative lung adenocarcinoma showing the efficacy of crizotinib. ROS1-rearranged primary lung cancer should be treated with an ROS1 inhibitor because of its proven efficacy; however, the efficacy of ROS1 inhibitors for ROS1-rearranged putative lung adenocarcinoma is uncertain. A prospective study is required to evaluate the efficacy of ROS1 inhibitor therapy for ROS1-rearranged CUP.

Various mutations in the gene that causes cancer have been discovered with the progress of genetic analysis technology. Efficacy of molecular-targeted medicine for cancers with driver oncogenes, such as HER-2 amplification in breast cancer, EGFR mutation and ALK-rearrangement in NSCLC, and BRAF mutation in malignant melanoma, is found to be very high. So far, we could detect only one mutation in the gene on one test. Recently, comprehensive mutation screening tools, such as NCC OncoPanel and OncoPrime, have become available [6, 7]; this will lead to further developments in precision medicine for cancer patients.

Recently immune checkpoint inhibitors are very important for the treatment of primary lung cancer. If PD-L1-TPS of lung cancer is ≥50%, first-line treatment with pembrolizumab, an immune checkpoint inhibitor, has improved the survival of patients with primary lung cancer [8]. Therefore, PD-L1-TPS examination is important to determine the best treatment for primary lung cancer. In only 22% of CUP, tumor PD-L1 expression was detected [9]. We previously reported that in EGFR mutation-positive putative lung adenocarcinoma presenting as CUP, high PD-L1 positivity (TPS: 80%) was detected [10]. In the present case, very high PD-L1 positivity (TPS: 100%) was detected. Putative lung adenocarcinoma with driver oncogenes might be a population with a high frequency of high-PD-L1-positive cases; however, the efficacy of immune checkpoint inhibitor therapy for these cases is unknown. A prospective study is desired to confirm the efficacy of immune checkpoint inhibitors for putative lung adenocarcinoma with or without high-PD-L1 positivity.

In conclusion, we report a case of ROS1-rearranged putative lung adenocarcinoma presenting as CUP showing good response to ROS1 inhibition therapy. Driver oncogenes, such as ROS1 rearrangement, EGFR mutation, and ALK rearrangement, should be examined in putative lung adenocarcinoma similar to that in primary lung adenocarcinoma.
